# Sperm, Clinics, and Parenthood

**DOI:** 10.1111/bioe.12270

**Published:** 2016-08-15

**Authors:** Reuven Brandt

**Keywords:** reproductive ethics, sperm donation, parenthood, gamete donation, HFE Act

## Abstract

In this article I examine a recent approach to regulating assisted reproduction, whereby use of some kind of medical intervention ‘triggers’ laws governing legal parenthood that are more favourable to intending parents and sperm providers. I argue that although perhaps an improvement on the previous legal framework, these laws are problematic for three important reasons. First, they are prone to violating parental rights and unjustly imposing substantial burdens on individuals. Second, they are discriminatory. Third, even if we take a pragmatic approach to the question of parenthood in these cases, these laws fail to properly consider the welfare interests of children. Finally, I conclude by showing that my argument does not entail adopting a laissez‐fair attitude to conception using third‐party sperm.

## 1 Introduction

Many jurisdictions have enacted legislation specifically governing the ascription of legal parenthood in cases where pregnancies occur by use of third‐party sperm.^1^ One legislative approach, the focus of this article, is to depart from the weight common law normally places on genetic ties by making intending parents who use third‐party sperm a child's sole legal parents, but only when conception occurs through use of some specified type of medical intervention. I will call legislative approaches that have this effect ‘modified parenthood by medical intervention statutes**’** (MPMIS). As a consequence of MPMIS, it is not only the consent and intentions of the sperm provider and intending parent(s) that the legislation takes into consideration when determining how to ascribe legal parenthood; whether conception occurs via the right kind of medical intervention plays a substantive role in determining which individual(s) the law recognizes as a child's legal parent(s). One example is the current regulatory framework in the United Kingdom, where the rules governing legal parenthood following use of third‐party sperm are different for individuals who are not married or in a civil partnership if conception takes place under the auspices of a medical clinic licensed to provide fertility treatments.^2^

In what follows I will argue that MPMIS are morally problematic for three principal reasons. First, they are prone to both (a) ascribe legal parenthood in ways that violate moral parental rights, and (b) unjustly burden individuals with weighty financial obligations. Second, they discriminate against certain reproducers by requiring them to undergo medical scrutiny that is not required of other reproducers. Lastly, the status quo cannot be defended by arguing that the law must take a pragmatic stance to the question of legal parenthood because there is uncertainty about what moral facts ultimately determine parenthood.

I will begin by briefly describing two legal decisions where medical intervention was relevant for determining which adults were a child's legal parents, and offer these as initial motivation for thinking that the legislation that led to these decision**s** is problematic. In the second section I will explain why MPMIS came about, their potential benefits, and why they are ethically suspect. In the third section I will briefly explain why my view does not entail a laissez‐faire approach to reproduction via third‐party sperm.

Although one of the examples I present is a case from the US, my discussion will focus on the law in the UK, and the Human Fertilization and Embryology Act (HFE Act) 1990 & 2008 in particular. This is because the development of the law in the UK was done in a relatively transparent fashion that provides some degree of access to the motivations behind the legislation. Despite my focus on UK law, I take the ethical analysis to apply to similar legislation elsewhere.

Throughout the text I will use the subscript _m_ to distinguish moral conceptions of parenthood, parental rights and parental responsibilities from their legal analogues.

## 2 Cases

### Bruce v Boardwine

The Court of Appeals of Virginia awarded Robert Boardwine custody and visitation rights for his genetic child following a private sperm donation arrangement that broke down.^3^ Boardwine had provided sperm to his friend, Joyce Bruce, with the understanding that Bruce would be the sole parent of the child. Bruce then inseminated herself with Boardwine's sperm using a turkey baster. However, following a dispute with Bruce, Boardwine changed his mind about the relationship he wanted with his genetic child and petitioned the court to recognize his parental rights. In its decision, the court found that a Virginia statute^4^ excludes sperm donors from legal parenthood only when insemination occurs with the aid of medical technology. Since Bruce used a turkey baster, which the court found does not satisfy the definition of ‘medical technology', Boardwine could not be legally treated as a sperm donor. Reaffirming the decision of the lower court, the judge therefore granted Boardwine access to his genetic child. Importantly, had conception taken place using medical technology rather than a turkey baster, the original agreement (which was not disputed by either party) could have been upheld by the court.

### Langridge

In the UK, Mark Langridge reported that he was ordered to pay child maintenance by the Child Support Agency over a decade after privately providing sperm to a lesbian couple.^5^ Though the children's birth mother disputed that she and Langridge had agreed that Langridge would be a mere sperm provider, this factual question was rendered immaterial by the legislative framework governing legal parenthood at the time. The HFE Act 1990 (which will be discussed in greater detail below) excluded sperm providers from legal responsibility for their genetic offspring only when sperm is transferred through a licensed medical clinic.^6^ Consequently, Langridge was the legal father of the children created using his gametes, despite any prior agreement between himself and the children's mother. However, had Langridge donated sperm through a licensed medical clinic, he would not be a legal parent of the resulting children (unless he had explicitly entered into a parenthood agreement with the intending mother), and thus would not have child maintenance obligations.

### Discussion

Even at first blush the importance placed on the use of medical intervention in these cases seems strange, since it is not immediately clear why technical details regarding the method of conception ought to be relevant for determining which individuals have parental rights and obligations for particular children. This is most acute in the Boardwine case, where the type of device (a turkey baster) used to deliver sperm was central to the court's finding that Boardwine was a legal parent. However, even in the Langridge case, it might seem odd that legal parenthood depends, in part, on whether a type of medical intervention (use of licensed healthcare provider) is involved in the fertilization process. As these cases demonstrate however, MPMIS make these factors relevant to legal parenthood. In what follows I will explain what brought about MPMIS, and why they are indeed morally problematic.

## Examination of MPMIS

### Parenthood Under Common Law

MPMIS arose, in part, out of the realization that parenthood under common law poorly accommodates reproduction via third‐party sperm because of the importance traditionally placed on biological ties when determining legal fatherhood. Though the relationship between genetic kinship and legal fatherhood is not strict, it is a factor that is given considerable weight, and in many cases is sufficient for legal parenthood.^7^ Perhaps the clearest example is unintended pregnancy. Consider a single woman who gives birth to a child conceived during a one‐time tryst. Despite the absence of any ongoing relationship with the child's mother, or any intention to parent a child, the genetic father is generally deemed the child's legal parent. In fact, in a case where a clinic accidentally used the sperm from the wrong man when treating a couple who were seeking to reproduce using their own gametes, the High Court ruled that the man whose sperm was accidentally used (and not the intending father) was the resulting children's legal father.^8^ In the case of sperm the law places such enormous weight on genetic ties that courts have even found men to be legal parents in circumstance where their sperm was purloined via fraud, and even when they were the victim sexual assault.^9^

When applied to assisted reproduction, this importance placed on genetic ties gives rise to worries for both sperm providers and individuals who wish to create families using third‐party sperm. For sperm providers, the worry is being deemed a legal parent of the child created using the provided gametes, as exemplified in the Langridge case described above. For individuals making use of third‐party sperm, the worry is that they risk having their family life disrupted by the parental claims of a sperm provider, as exemplified in the Boardwine case. Though in cases where a woman is married there is a common law presumption that her spouse is the child's legal parent, this presumption can be rebutted by genetic tests requested by either the presumed father, or another man who asserts a claim to paternity.^10^ In earlier decisions, courts were hesitant to permit contestations of paternity when they risked disrupting a functioning family unit.^11^ However, at least in the UK, there has been a shift towards determining a child's legal paternity on the basis of genetics once a challenge is made,^12^ despite potential disruptions to the existing family structure that might arise as a consequence.^13^

The traditional legal approach to parenthood thus raises several problems for individuals wishing to reproduce using third‐party sperm. As in the Langridge case, it puts sperm providers at risk of being deemed the legal parents of their genetic children, which brings with it significant obligations, despite their intentions when providing sperm. As in the Boardwine case, it puts intending parents at risk of having sperm providers granted the legal authority to interfere with their private family life.

### Purpose of MPMIS

MPMIS arose to create a framework specifically governing reproduction via third‐party sperm that is more favorable to the interests of intending parents, gamete providers, and arguably their offspring,^14^ than legal parenthood under common law.^15^ For instance, in The Report of the Committee of Inquiry into Human Fertilization and Embryology (hereafter referred to as ‘The Warnock Report’) which laid much of the conceptual groundwork for the HFE Act 1990, the committee recommended that reproduction via third‐party gametes be accepted as legitimate treatment for infertility, and that third‐party sperm providers not have any legal rights or responsibilities in respect of the children that result from their gametes.^16^ The committee's recommendation was an explicit call to depart from the consequences of reproduction via third‐party sperm under common law, in favour of legislation that treated such procedures as assisted reproduction for intending parents. To this effect the committee stated:

Clearly in view of our recommendation (4.17) that the AID child should for all purposes be treated as the legitimate child of the couple who have benefitted from successful treatment, the donor should have no responsibilities towards the child. We therefore recommend a change in the law so that the semen donor will hnave no parental rights or duties in relation to the child.^17^

A similar recommendation was made in respect of ova donors, though in this case it affirmed the common law approach that gestation and not genetics should determine motherhood.^18^

That the Warnock Report recommended altering the conditions that give rise to legal parenthood does not fully explain MPMIS because this suggestion alone in no way implies that use of specially licensed clinics ought to have any bearing on how courts determine legal parenthood. At least in the UK, the key motivation for including licensed clinics as part of the framework governing parenthood, which in turn motivates MPMIS, was to ensure the health and safety of the individuals involved, including the children who would eventually result from third‐party gametes. For example, later in The Warnock Report the committee states that they see ‘the protection of the public…as the primary objective of regulation’ and that they ‘believe that all the techniques require active regulation and monitoring, even though, as we realise, such restrictions may be regarded by some as infringing clinical or academic freedom’.^19^ Under the HFE Act 1990, such measures include limiting the number of donor offspring in order to prevent accidental consanguineous relationship, screening donors and their gametes in order to prevent the transmission of diseases, and screening both donors and recipients in order to ensure that they are medically fit to undergo their respective procedures.^20^ MPMIS thus rest on the dual concerns that the common law does not adequately accommodate reproduction via third‐party sperm and that the state has an interest in ensuring that any such reproduction is done in a safe manner.

We might reasonably think that The Warnock Report's proposal is a welcome one. First, it creates a mechanism for individuals reproducing by means of third‐party sperm to become legal parents from the moment of birth, while also excluding third‐party sperm providers from later gaining parental status. That third‐party sperm providers are excluded from being legal parents also means that they do not face the possibility of being burdened with child maintenance obligations. Second, the medical requirements that clinics are obligated to fulfill protect the health interests of the individuals seeking treatment and any children that may result. However, in the following section I will argue that coupling the modified criteria for legal parenthood to the clinical requirements is in fact morally problematic. I will argue that, at best, MPMIS discriminate against individuals reproducing via third party sperm by unjustly *requiring* them to undergo clinical procedures and screening that are not required of other reproducers if they want to ensure legal recognition as the child's parents to the exclusion of sperm providers.^21^ At worst, MPMIS are prone to violating moral parental rights, and imposing unjust financial burdens on those proving their sperm for the reproductive use of others.

### MPMIS and Harm

#### Moral Rights and MPMIS

The purpose of MPMIS is to codify the ascription of legal parenthood, and presumably we want this ascription to track the facts that are relevant to moral parenthood in so far as this is possible. Ideally, legal parents would be moral parents (parents_m_), and vice‐versa. When we consider the normative features of parental_m_ rights, two important questions to be addressed are, (1) what is the content of parental_m_ rights? and (2) who are the holders of these rights? A particular theory regarding what ultimately grounds parental_m_ rights might have consequences for each of these questions.^22^ For instance, individuals who take a strong child‐centric approach to parental_m_ rights might think that it is the individual(s) best suited to promote the interests of the child that have parental_m_ rights, and that these rights are constrained by the obligation to always act in the child's best interest. Alternatively, individuals who take a more parent‐centric approach might think that the interests of a person or people reproducing with the aim of parenting play a significant role in determining who has parental_m_ rights, but that these rights are limited to a certain extent by the interests of the children they parent.^23^ Turning to the first question, though the philosophical literature is very much divided on the precise content of parental rights, authors from both parent‐centric and child‐centric approaches broadly agree that parents ought to have a certain degree of privacy and autonomy when raising their children.^24^ Outside philosophical debates about parenthood, the right to private family life is also considered important, as evidenced by its inclusion in the European Convention on Human Rights,^25^ The Human Rights Act^26^ and the UN's Universal Declaration of Human Rights.^27^ Although defending a particular view about the normative extension of the right to private and autonomous family life is beyond the scope of this paper, there are examples that I take to be relatively uncontroversial. Consider, for instance, a family that plans to take their young child on a road trip to celebrate a cultural festival with their extended family. Although a neighbouring family might like to include the infant in their family event, and might even make the case that the event they propose will be more beneficial to the young child, we still think that the child's parents have the right to take their child to their family's celebration.

The second question, identifying who is the holder of parental_m_ rights, is equally controversial. In the philosophical literature, four kinds of theories dominate the debate: genetic accounts,^28^ gestational accounts,^29^ intentional accounts,^30^ and causal accounts.^31^ According to the genetic account of parenthood, individuals acquire parental_m_ rights because their progeny's genetic material is derived from their own. According to intentionalists, parental_m_ rights arise from the intention to parent a child. On the gestationalist account, parental_m_ rights arise primarily from the act of gestation, and others’ parental claims or status as a moral parent arises from their social relationship with the gestating woman. Lastly, causal accounts of parenthood determine parental_m_ rights on the basis of the kind of causal role individuals have in bringing a child into existence. What is important for the purposes of this discussion is that the involvement of a licensed clinic plays no role in determining parental_m_ rights in any of the major accounts. Use of licensed clinic does not alter: the genetic link between reproducers and their offspring, the intensions of the involved parties, or the identity of the person who gestates a child. And although some causal theorists might include clinic employees as individuals who have some responsibility towards the children they help create,^32^ that the clinic is licensed is irrelevant to the nature of the causal role they play in bringing a child into existence. So while causal theorists might conclude that the involvement of additional parties in the creation of a child, such as medical professionals, affects the structure of the responsibilities various adults have towards a child, it seems unlikely that being a *licensed* professional is of any importance to the causal relationships from which responsibility is ultimately derived.

It is therefore reasonable to conclude that the presence or absence of licensed medical treatment is unlikely to determine which individuals have parental_m_ rights and responsibilities for particular children. Individuals who have an intentional or gestational view of parenthood_m_ will think that granting legally protected contact to a sperm provider who changes his mind about the kind of relationship he wishes to have with the child he helped to create, merely because conception occurred outside the auspices of a licensed clinic, violates the parental_m_ rights of the commissioning parent(s) and/or gestating parent. Similarly, individuals who have a genetic or causal view of parenthood will think that refusing to recognize a sperm donor as a legal parent merely because a licensed clinic was used violates the sperm provider's parental_m_ rights.^33^ Consequently, MPMIS will assign legal parental rights and responsibilities in ways that diverge from their moral analogues, at least in some cases. This in turn will result in some individuals being denied their parental_m_ right to privacy and autonomy in raising their children, regardless of which theory of parenthood_m_ is adopted.

A similar argument also arises concerning the legal obligations that follow from legal parenthood. In a recent case in the UK, a private sperm donor who was found to be a legal parent claimed that his financial obligations to his genetic offspring were preventing him from starting a family of his own.^34^ And as noted by Elizabeth Brake, child maintenance obligations can result in substantial burdens, so imposing them ought to done in an ethically acceptable fashion that considers the rights and interests of all parties.^35^ Determining exactly what kind of involvement in procreation brings about a moral obligation to provide child maintenance is as contentious as determining what kind of procreative involvement results in parental rights. However, as in the case of parental rights, it is unlikely that the use of a licensed clinic will play any role in determining which individuals have a moral obligation to support the children they have helped to create. Thus, just as MPMIS risk violating parental rights, they also risk either burdening individuals with child maintenance obligations in a morally problematic way, or legally ‘letting off the hook’ individuals who have the moral obligation to support certain children.

One defence of cleaving legal parenthood from moral parenthood in these sorts of cases, as I have argued, occurs as a result of MPMIS, might be that we are justified in departing from the moral facts governing parenthood if it is to encourage other important moral ends, like the health of the involved parties and of future generations. As was noted previously, the justification offered in the Warnock Report for regulating assisted reproduction was the health and safety of the involved parties. Although sperm provision is not a procedure with many inherent risks, we might think that MPMIS are an important tool for ensuring that IVF and other similar procedures are done safely, given the significant health complications that are possible. We might also think that MPMIS are important for ensuring that: (a) women undergoing treatment are sufficiently healthy to undergo pregnancy, (b) the gametes do not pose a risk of infectious disease to the woman gestating or the resulting child, and (c) the risk of passing congenital diseases to the resulting children and future generations (including through accidental consanguineous relationships) is minimized.

However, similar potential health risks arise from sperm in traditional sexual reproduction, and yet there is no similar regulatory framework in place that encourages reproducers to minimize those risks.^36^ Individuals reproducing sexually risk acquiring infectious diseases, passing infectious diseases to their offspring, and passing congenital diseases to their offspring. Crucially, there is no need for individuals wishing to become parents through sexual intercourse to proceed in a manner that meets some health and safety standard in order for the law to grant them legal parental rights and responsibilities. This is the case even when individuals wilfully engage in high‐risk activities, like reproducing with a known carrier of an autosomally dominant genetic disease, or sexually transmitted infection. Thus, even if we think that encouraging responsible reproductive practices justifies assigning legal parenthood in a manner that departs from parenthood_m_ we have a problem of parity: the law only takes these welfare factors into consideration when individuals reproduce using third‐party sperm. As will be discussed in more detail in the following section, this raises a problem of discrimination against individuals reproducing in this way.^37^

One possible explanation for this asymmetry might be that assisted reproduction necessitates medical intervention in a way that sexual reproduction does not, and so we might think that MPMIS are primarily about setting standards for medical procedures and thus are not that unusual. However, there are several reasons to reject this explanation. First, as the case Bruce v Boardwine discussed above exemplifies, artificial insemination by donor (AID) is not a particularly technical procedure that necessitates medical intervention. Though we might think that this is an unforeseen exception to the general framework of the HFE Act 1990, the Warnock report does explicitly note that ova donation is importantly different from AID in that ova donation necessitates medical intervention in a way that AID does not, yet still concludes that all procedures ought to be regulated.^38^ Second, it is unclear why failure to respect medical guidelines ought to have any bearing on how legal parenthood is determined. Presumably medical guidelines are in place largely for welfare reasons, and so if we think that an individual risking her own or her future child's welfare has no bearing on legal parenthood in the case of sexual reproduction, it is unclear why similar risk‐taking ought to play a role in legal parenthood when individuals reproduce via third‐party sperm.

I would like to emphasize that my argument up to this point *is not* merely that MPMIS rely on an ethically contentious theory of parenthood_m_ as a basis for assigning legal parental rights and obligations. Rather, my argument is that MPMIS take no morally principled stand whatsoever on the features that give rise to parental_m_ rights and responsibilities. Consequently, MPMIS will result in violations of parental_m_ rights and unjust impositions of weighty burdens *regardless* of the account of parenthood_m_ that is adopted. Furthermore MPMIS cannot be defended on the grounds that they promote the heath interests of reproducers, their children, and society at large, because these concerns are not considered in other instances of reproduction, despite similar risks.

#### Discrimination

Despite the theoretical problems discussed above, individuals who endorse an intentionalist account of parenthood, at least in gamete provision cases,^39^ might somewhat approve of MPMIS. Although MPMIS require reproducers wishing to become legal parents using third‐party sperm to undergo greater medical scrutiny, they at least provide a route to guaranteed legal parenthood for certain parents_m_ where none was available before. While this might make MPMIS an improvement to the previous legal framework (depending on one's view of moral parenthood), it does not mean that they are free of serious moral problems. **C**onsider the following analogy.

We could imagine a state in which a body of law developed such that inter‐racial couples were incapable of being recognized as a child's legal parents, regardless of whether a child was a product of their gametes. We could further imagine that, after much public consultation and debate, the state decided it was time to amend the law to include inter‐racial couples as legal parents of the children they create through sexual reproduction, despite looming questions about the ethics of inter‐racial parenthood. However, under the new law, inter‐racial couples would be required to first undergo medical screening and be certified ‘safe’ or the previous law denying them legal parenthood would apply. For the same‐race couple, no screening would be required. Although we might think that this law is an improvement to the preceding legal framework, we would still think it was problematically discriminatory. So if we think that individuals who use third‐party gametes to reproduce, such as same‐sex couples, infertile individuals, and infertile heterosexual couples, should be just as much parents from the moment of birth as intending parents who reproduce using their own gametes, then we have reason to find MPMIS problematic.^40^

There is however a difference between assisted reproduction and the inter‐racial case that might make MPMIs seem more palatable. In the case of the inter‐racial couple, part of what makes the modified legal framework seem so unacceptable is that the identity of the parents_m_ is clear, and so including them as legal parents could be easily accomplished with a legislative change. In the case of reproduction by third‐party gametes, circumstances might arise where it becomes difficult to determine which individuals the law should recognize as a child's parents, even if there is agreement about what approach the law should take when determining parenthood. Disputes might arise about the nature of the sperm‐provision arrangement. For instance, a man who has intercourse with a woman with the understanding that they would co‐parent any resulting children might, during the course of the pregnancy, change his mind about wanting to parent. As a means of escaping child maintenance obligations, he might later claim that his genetic child was actually the product of a third‐party sperm provision agreement.^41^ Alternatively, a sperm donor might change his mind might about the relationship he wants to have with his genetic child, and later deny that he provided sperm with the understanding that he was not to be a parent. Unlike in the inter‐racial example, we are therefore left with a pragmatic problem: we require a method for distinguishing private third‐party sperm provision agreements from reproduction where the genetic parents are also the legal parents. Someone might think that MPMIS are acceptable because they resolve this problem by creating clear guidelines for establishing that a third‐party sperm provision agreement was in force.

The question that looms large in these cases is where to place the burden of proof, since at some point the state must determine which individual(s) have legal rights and responsibilities for a child. For reasons that I will not fully articulate here,^42^ it might makes sense to presume that legal parental standing should follow the common law approach, barring conclusive evidence that there was an agreement to depart from this norm. In this vein, use of a licensed clinic ought to be strong evidence, if not definitive proof, that such an agreement was in fact in place. But the need to resolve these kinds of disputes cannot justify MPMIS, because although use of a clinic is probably sufficient for refuting a presumption in favour of genetically determined parenthood, it should not be necessary. For instance, I see no reason why the law could not be amended so that a signed and notarised document outlining the intentions of the involved parties would suffice to outweigh any presumption in favour of biological parenthood. Given the lifelong and serious implications for intending parents, children, and individuals who intended to be mere sperm providers, determining legal parenthood ought not to rest entirely on a single point of procedure that imposes more burdens on some reproducers than others. This is especially the case when the individuals most likely to be affected, like same‐sex couples and intending single parents, have historically been discriminated against when seeking to become parents.

Permitting individuals to demonstrate that they have entered into a sperm provision arrangement without requiring them to seek the services of medical professionals is not unprecedented. Under current UK law this is in fact possible in certain circumstances. Individuals who are in a civil partnership or are married and who reproduce using third party gametes become the legal parents of any resulting children to the exclusion of the gamete provider, so long as they can establish (a) that a gamete provision agreement was made prior to conception and (b) the civil partner/spouse of the woman receiving treatment consented to the use of third party gametes.^43^ Other jurisdictions apply this approach more generally. For example, the Family Law Act in British Columbia requires only that some agreement making clear the intentions of the involved parties be made prior to conception in order that a pregnancy to be legally considered the result of assisted reproduction.^44^ In such cases, legal parenthood goes to the intending parent(s), regardless of genetic relatedness and regardless of whether conception occurs under the auspices of a medical clinic.^45^ Under this model, cases like Boardwine's would not arise because the agreement would be enforceable even if insemination took place using a turkey baster.

#### MPMIS on pragmatic grounds

If we take moral uncertainty about parenthood_m_ as a background condition, we might think that the best any legislation could do is offer a pragmatic solution in the face of competing views about parental rights and responsibilities. This is because although there is philosophical disagreement about what makes an individual a parent_m_, the law does not have the luxury of waiting for the dust to settle, and must adjudicate disputes about parenthood in a timely fashion. This possible defence of MPMIS on pragmatic grounds might proceed as follows. In cases where we are relatively certain about determinations of parenthood_m_, keeping legal parenthood consistent with the moral facts ought to be prioritized.^46^ In cases where determining parenthood_m_ is less clear, we are justified in crafting laws that assign legal parenthood in ways that promote other important ends, such as the health of reproducers, their children, and future generations. We might think that focussing on promoting these other goods is an acceptable aim of policy because, due to uncertainty about parenthood_m_, ensuring that parental_m_ rights are protected is not possible. On this view, then, MPMIS are justified because they discourage individuals from reproducing in risky ways; the consequence of not following the proper medical route that minimizes negative health consequences is having legal parenthood assigned in a manner that is not in keeping with the stated intentions of the involved parties.

A major problem with this defence of MPMIS, however, is that it considers only the benefits of discouraging risky reproductive behaviour, and fails to adequately take into consideration the future welfare interests of the children to be parented. Although it may be true that the state should take measures to encourage individuals to reproduce in a responsible manner, it is far from clear that this aim ought to determine who has rights and obligations towards a child. Consider the following hypothetical case. A woman decides to reproduce using third‐party sperm, but does not want to go through the hassle of using a licensed clinic. Instead, she illegally purchases sperm from a self‐described ‘party animal’ who has neither the financial means nor the disposition to adequately parent a child. Because she does not use a licensed clinic, the state later determines that the sperm provider is one of the child's legal parents.

Even if we presume uncertainty about parenthood_m_, it is still intuitively inappropriate for the state to discourage this kind of ‘black market’ sperm provision by making the sperm provider one of the child's legal parents, rather than mother's partner/spouse. This is because doing so worsens the resulting child's predicament, which seems wrong because the child is an unconsenting innocent party to the arrangement made between the couple and the sperm provider. Not only are the child's health interests put at risk because unscreened sperm is used, but her family life is rendered prone to interjection^47^ from an individual far less suitable to parent. A more reasonable approach in circumstances in which (a) there is uncertainty about parenthood_m_ and (b) procedures put in place to regulate assisted reproduction were not followed, is to decide parenthood on the basis of the best interests of the child.^48^ This approach would discourage ‘black market’ transactions by discounting the interests of reproducers in favour of those of the children they create, while also ensuring that children are not further harmed by the state's attempt to promote more careful reproductive practices.

### Discussion

I have argued that MPMIS are morally problematic for three major reasons. First, they risk assigning weighty legal rights and obligations in ways that depart from the underlying moral facts. Importantly, this argument does not rest on taking a position on which parties in a third‐party sperm provision case ought to be the child's legal parents. Second, even if we think the state should provide some mechanism for accommodating intentional parenthood when individuals reproduce using third‐party sperm, MPMIS are not justified. This is because MPMIS impose medical scrutiny as a condition of legal parenthood, which is a burden not imposed on other reproducers, and one that is not needed for determining whether a third‐party sperm provision agreement was in place. Finally, MPMIS cannot be defended on the grounds that in the absence of relative certainty about parenthood_m_, crafting laws about parenthood that promote goods other than protecting parenthood_m_, like promoting more health‐conscious reproductive practices, is justified. This is because MPMIS fail to adequately take into consideration the welfare interests of the existing child and instead focus on ‘punishing’ the adults who violate regulations governing reproduction. In closing I will briefly discuss what implications this argument has for the regulation of third‐party sperm provision more generally.

## 3 Should Regulation be Abandoned Completely?

The preceding argument might be interpreted as advocating for a relatively laissez‐faire attitude towards reproduction via third‐party sperm, if it is to be permitted at all. Since I argue that there is no good justification for requiring individuals reproducing using third‐party sperm to use a licensed clinic in order to be recognized as legal parents, someone might think that my view entails opening the floodgates to all kinds of unscrupulous practices. We might envision unregulated IVF practitioners that place little weight on the safety of their patients, and instead focus on providing low‐cost and high‐profit services. However, my argument is much narrower.

First, I argue that there are no good justifications for making the promotion of health‐conscious reproductive practices relevant to legal parenthood when reproduction occurs via third‐party gamete provision, but not in traditional reproduction. If a strong argument could be made that promoting heath was more important than having legal parenthood track parenthood_m_, and society adopted heath‐screening requirements for all reproducers, then having legal parenthood in third‐party gamete cases depend on health screening would not be problematic. Second, if we do not think that promoting health‐conscious reproductive practices ought to be relevant to determinations of legal parenthood in traditional reproduction, which is the stance currently reflected in law, my argument merely implies that the same should be true in third‐party gamete cases. This implies nothing about consequences for violating regulations governing assisted reproduction that do not impinge on legal parenthood. For instance, we might still think that providing IVF services without a licence constitutes practising medicine without a licence and ought to be criminal. We could similarly think that storage and distribution of sperm ought be done in accordance with strict safety guidelines, and fine or imprison individuals who violate these regulations. This approach would be consistent with how we currently treat intending parents who use their own sperm when reproducing, but might otherwise violate medical regulations. For instance, acquiring and using restricted fertility drugs without a prescription violates health regulations, even if the individuals making use of the drugs reproduce using their own sperm. While we may punish individuals who violate health regulations in this way, the consequences they face do not include alterations to how legal parenthood is determined. My argument therefore leaves open room for regulating assisted reproduction, but shows that violations of these regulations should not rule out intending parents as legal parents or automatically include gamete providers as legal parents. So although someone might think that individuals using third‐party gametes who reproduce with a turkey baster rather than following the mandated procedure should face some consequences (and I am not suggesting there should), my argument shows that legal parenthood should not necessarily lie in the balance.

## Footnotes

1
By third‐party sperm I mean sperm provided to intending parents with the understanding that the sperm provider will not be the parent of any resulting children. I find this terminology better than ‘donor sperm’ because the argument here applies equally to sperm that has been commercially acquired.

2
Human Fertilization and Embryology Act 2008; Human Fertilization and Embryology Act 1990.

3
Court of Appeals of Virginia, Record No. 1250–14‐3 (Bruce v Boardwine).

4
Code of Virginia. Title 20, Chapter 9.

5

M Brignall . 2012 Gay sperm donor told to pay child maintenance for 'his' two children. The Guardian. 26 October.

6
Note that later amendments arising from the HFE Act 2008 allow for some exceptions to this rule. See the discussion at the bottom of page 18 and footnote 43.

7
For a good discussion on tensions that arise in legal approaches to fatherhood see: A. Bainham. 2004. Contact as a Right and Obligation. In Bainham, Lindley, Richards and Trinder, ed. *Children and Their Families*. Portland: Hart Publishing: 61–88.

8
Leeds Teaching Hospitals NHS Trust v A & Ors [2003] EWHC 259 (QB). 16998996

9
For examples and discussion see: S. Sheldon. ‘Sperm bandits’, Birth Control Fraud and the Battle of the Sexes. *Legal Studies* 2001; 21: 460–480; and D. Benatar. 2012 *The Second Sexism: Discrimination Against Men and Boys*. Chichester: John Wiley & Sons: Chapter 3.

10
Currently such a rebuttal is possible only if the civil partner/spouse of the woman seeking treatment did not consent to the treatment. HFE Act 2008. *op. cit*. note 2. Sec. 35 and 42;

11
L. Morgan. Child Support Fifty Years Later. *Family Law Quarterly* 2008; 42(3): 365–380. p. 373.

12
In Re H, (Blood Tests: Parental Rights) [1997] Fam 89. It is also worth noting that there has been some push in the other direction. See L v P (Paternity Test: Child's Objections) [2011] EWHC 3399.

13
It is worth mentioning that though the common law places great importance on genetic ties when determining legal fatherhood, this is not so in the case of motherhood. There is a common law presumption that the gestational mother is a child's legal mother, regardless of whether she was the source of ova. This presumption possibly arose due to the fact that until recently the woman gestating was also always the ova provider, and so there was no impetus for the law to distinguish between the two kinds of contribution when determining a child's legal mother. Many jurisdictions (including the UK) have codified this presumption (HFE Act 2008, sec. 33), though it is not without controversy. Some scholars have pointed out that if the contribution of gametes alone suffices for men to be a legal parents, then it is unclear why the same should not hold for women as well.[T. Bayne & A. Kolers Towards a Pluralistic Account of Parenthood. *Bioethics* 2003; 17(3): 221–242.] There is room for much ethical debate about the relative weight that should be afforded to different types of reproductive contributions when resolving conflicts over legal parenthood and about the structure of legal parenthood more generally, but as it stands it is gestation and not the provision of ova that determines motherhood in the UK.

14
The HFE Act 1990 requires that clinics consider the welfare interests of future and existing children when determining whether to provide fertility treatment to intending parents. See Human Fertilization and Embryology Act 1990 sec. 13 (5).

15
This move is not uncontroversial, and some legal scholars have argued that that legal parenthood should track biological ties as far is possible. For instance Andrew Bainham states that, ‘The concept of parentage should rather be confined, to reflect as far as possible the unique position of biological parents and, through the child's filiation with them, the wider kinship links to the extended maternal and paternal families’. A. Bainham. Arguments about Parentage. *Camb Jaw J* 2008; 67(2): 322–351. p. 349.

16
Committee of Inquiry Into Human Fertilisation and Embryology Report. Committee of Inquiry Into Human Fertilisation and Embryology Report Her Majesty's Stationary Office, London (1984) (July) Sec. 4.17; Sec. 4.22; Sec. 6.8.

17
Ibid: Sec. 4.22.

18
Ibid Sec. 6.8.

19
Ibid. Sec. 13.1; Sec. 13.3.

20
See Human Fertilization and Embryology Code of Practice, 8^th^ Edition. 2015; Sec 11.; E. Jackson. 2015. The law and DIY assisted conception. In *Revisiting the Regulation of Human Fertilization and Embryology*. K. Horsey, ed. New York: Routledge: 31–49.

21
For women using donated gametes, being a legal parent is not the problem but, as in the Boardwine case, they risk having the sperm provider gain status as the child's legal parent. This might cause serious disruptions to the woman's private family life.

22
H. Brighouse & A .Swift. Parents’ Rights and the Value of the Family. *Ethics* 2007; 117(1): 80–108.

23
Ibid.

24
For a child‐centric approach see S. Hannan & R. Vernon. Parental Rights: A Role‐Based Approach. *Theory and Research in Education* 2008; 6(2): 173–189. For parent‐centric approaches see: F. Shoeman. Rights of Children, Rights of Parents and the Moral Basis of the Family. *Ethics*. 1980; 91(1): 6–19; Brighouse and Swift, Ibid.

25
Council of Europe. European Convention on Human Rights. Article 8.

26
The Human Rights Act, 1998.

27
United Nations General Assembly. Universal Declaration of Human Rights. Article 16.

28
For examples see JD. Velleman. Persons in Prospect. *Philos Public Aff* 2008; 36(3): 221–288.; JD. Velleman. Family History. *Philosophical Papers* 2005; 34(3):357–78.

29
For an example, see S. Feldman. Multiple Biological Mothers: The Case For Gestation. *J Soc Philos* 1992; 23(1): 98–104. 10.1111/j.1467-9833.1992.tb00488.x11659502

30
E. Brake. Fatherhood and Child Support: Do Men Have a Right to Choose? *J Appl Philos* 2005; 22(1): 55–73.; E. Brake. 2010. Willing Parents: A Volunturist Account of Parental Role Obligations. In. *Procreation and Parenthood*. D. Archard & D. Benatar, eds. Oxford: Oxford University Press: 151–177.

31
For examples see, J. Nelson. Parental Obligations and the Ethics of Surrogacy: A Causal Perspective. *Public Aff Q* 1991; 5(1): 49–61; G. Fuscaldo. Genetic Ties: Are They Morally Binding? *Bioethics* 2006; 20(2): 64–76. 11652011

32
L. Porter. How and Why to Prefer a Causal Account of Parenthood. *J Soc Philos* 2014; 45(2): 82–102. 10.1111/josp.12059PMC437317725821255

33
A similar argument also holds true for child‐centric defences of genetic parenthood. If, for example, we thought that genetic parenthood was defensible on the grounds that it is in the best interest of the child, then the status quo would still be problematic because it is unclear why this would cease to be the case when a licensed clinic is involved.

34
R. Williams. Sperm Donor to Lesbian Couple Forced to Pay Child Support. *The Guardian* 2007 4 December 2007.

35
Brake (2005) *op. cit*. note 30.

36
McLeod and Botterell raise similar concerns about regulations in adoption. See C. McLeod & A. Botterell. 2014. ‘Not For the Faint of Heart’: Assessing the Status Quo on Adoption and Parental Licensing. In *Family Making*. F. Baylis and C. McLeod, eds. Oxford: Oxford University Press: 151–167.

37
For a discussion of injustices caused by the invocation of welfare concerns in the regulation of assisted reproduction see: E. Jackson. Conception and the Irrelevance of the Welfare Principle. *Mod Law Rev* 2002; 65:176–203; E. Jackson. 2007. Rethinking the Pre‐Conception Welfare Principle. In *Human Fertilisation and Ebryology: Reproducing Regulation*. K. Horsey and H. Biggs, eds. Abingdon, Oxon: Routledge‐Cavendish: 47–68.

38
Warnock Report. *op. cit*. note 16. Sec. 6.6.

39
T. Bayne & A. Kolers. Towards a Pluralistic Account of Parenthood. *Bioethics* 2003; 17(3):221–42. 10.1111/1467-8519.0033814533607

40
See discussion about injustice in Jackson *op. cit*. note 37.

41
One solution is to take a strong intentionalist approach to parental responsibilities along the lines of Elizabeth Brake, and deny that the legal parenthood ought to necessarily arise in one‐night‐stand cases. See Brake (2005). *op. cit* note 30. and Brake (2010) *op.cit*. note 30.

42
For instance, given the historical importance placed on genetic ties, most people engaging in sexual reproduction do so with the understanding that responsibility for ensuing children will be shared between procreators. Given that this is already the default position, it makes sense that the burden of proof should be on demonstrating that the involved parties consented to depart from this norm.

43
That this option is not available to single people is problematic, especially given that single women are able to become the sole legal parents of a child if they use donor gametes through a licensed clinic. It is unclear why having a civil partner or spouse should replace any legitimate role that clinics play in the determination of legal parenthood, or why the consent of an individual not receiving treatment (the civil partner/spouse) is relevant to how the law determines legal parenthood; HFE Act 2008, sections 35 and 42.

44
British Columbia. Family Law Act. SBC 2011; Part 3.

45
Ibid.

46
We tend to think that moral parents ought to be given the opportunity to parent their children, unless there is strong evidence that they pose a serious threat to the future welfare of their children. For a discussion see, D. Archard. Child Abuse: Parental Rights and the Interests of the Child. *J Appl Philos* 1980; 7(2): 183–194.

47
For instance, the legal parent could petition for contact rights, or even shared residency. At a minimum, this would put the child through the ordeal of a series of legal proceedings, and if the petition is successful could result in the child having to spend significant amounts of time away from the more suitable parent, with someone whose influence may not be in the child's best interest.

48
This proposal raises two important questions which I cannot fully address in this article. One is determining the group of individuals that are appropriate candidates for being a particular child's parent(s). On this question, Bayne and Kolers’ paper on parental pluralism (*op. cit* note 13) might serve as good starting point. The second is how to determine what is in the best interest of a child. Here I suggest, as a good analysis, J. Herring. Farewell Welfare? *J Soc Welf Fam Law* 2005; 27(2): 159–171.

